# Conservative management of amlodipine influenced gingival enlargement

**DOI:** 10.4103/0972-124X.51894

**Published:** 2009

**Authors:** Rashmi P. Dhale, Mangesh B. Phadnaik

**Affiliations:** *Postgraduate Student, Department of Periodontics, Government Dental College and Hospital, Aurangabad – 431 001, Maharashtra, India*; 1 *Associate Professor and Head, Department of Periodontics, Government Dental College and Hospital, Aurangabad – 431 001, Maharashtra, India*

**Keywords:** Amlodipine, conservative approach, gingival enlargement

## Abstract

Gingival enlargement is a well recognized unwanted effect associated mainly with anticonvulsant drugs, immunosuppressant drugs and calcium channel blockers. Amlodipine influenced gingival enlargement is comparatively less prevalent amongst calcium channel blockers. It causes aesthetic disfigurement, speech disturbances, abnormal tooth movement and difficulty in mastication. The management of drug influenced gingival enlargement is a challenge for the periodontist, mainly due to less understanding of its pathogenesis, difficulties in selection of proper line of management and recurrence of the enlargement. This report discusses the importance of conservative approach (scaling and root planning along with drug replacement) in the management of a case of amlodipine influenced gingival enlargement. The need for extensive surgery was decreased after this approach.

## INTRODUCTION

Gingival enlargement is a well recognized, unwanted effect associated mainly with anticonvulsant drugs, immunosuppressant drugs and the calcium channel blockers. Severe gingival enlargement is often disfiguring and can interfere with speech, occlusion and mastication.

Calcium channel blockers are used in the management of arrhythmias, angina pectoris and hypertension. The subjects taking nifedipine appeared to be at more risk for developing enlargement than those taking amlodipine.[1] Mild hyperplasia was detected with amlodipine with a prevalence rate of 3.3%.[2]

The pathogenesis of drug influenced gingival enlargement suggest that it is multifactorial including; age, genetic predisposition, pharmacokinetic variables, drug induced alteration in gingival connective tissue homeostasis, plaque induced inflammatory changes and drug induced action on growth factors.[3]

It starts as a painless, beadlike enlargement of the interdental papilla and extends to facial and lingual gingival margins. It may partially or completely cover the tooth surfaces. If there is underlying periodontal disease then the tissues may appear inflamed.[4] It tends to be more severe in areas where plaque accumulates. Otherwise the gingival enlargement is distributed symmetrically and for the anterior teeth to be more severely affected than the posterior teeth.[5]

The diagnosis is mainly based on the medical history, clinical features and histopathological features.

The treatment of drug influenced gingival enlargement is a challenge for the periodontist due to difficulty in the selection of proper line of management and its high recurrence rate.[6]

Comprehensive treatment of these cases is multidisciplinary in nature. Dentists and physicians should first consider the nonsurgical approach, including the removal of local factors and replacement of the offending drug. If the nonsurgical approach is not effective, periodontal surgery can remove the enlarged gingival tissues.

This report emphasizes the importance of conservative treatment in the management of a case of amlodipine influenced gingival enlargement.

## CASE REPORT

A 35-year-old female patient reported with a chief complaint of “swollen gums”. A medical history of the patient revealed that the patient was hypertensive and taking antihypertensive drug, amlodipine (2.5 mg/day, single dose orally) since 42 months. The patient noted a gradual enlargement of the gingiva of 28 months duration. Dental history revealed that the patient had undergone scaling 18 months ago, after which there was only little reduction in the size of enlargement.

### Examination

**Figure 1:**
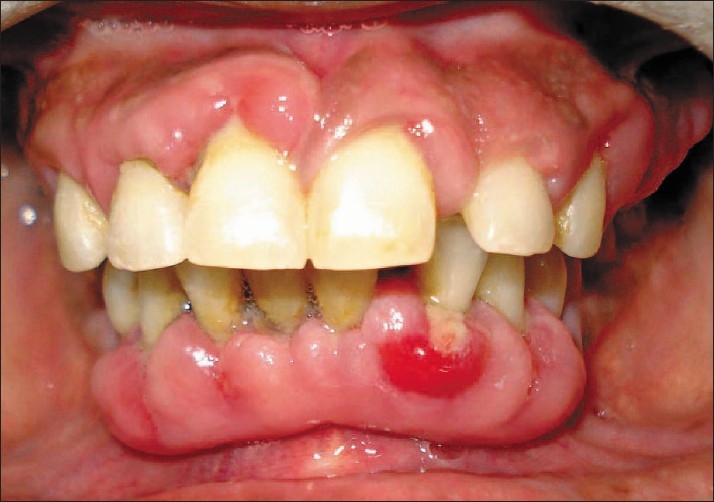
Front view at baseline

A generalized enlargement of the gingiva was present, more in the anterior region. The enlargement was more on the left side because of unilateral mastication from right side. Gingiva was reddish pink, soft and bled on probing. Suppuration was seen with 21. The mean pocket probing depth was 6.4 mm; mean plaque index was 2.4 and mean gingival index was 1.9. Grade III mobility with 26, grade I mobility with 31, 32, 41 and 42 was present. Proximal caries were seen with 17 and 18 [Figure 1].

### Investigations

Complete Haemogram was normal.

**Figure 2:**
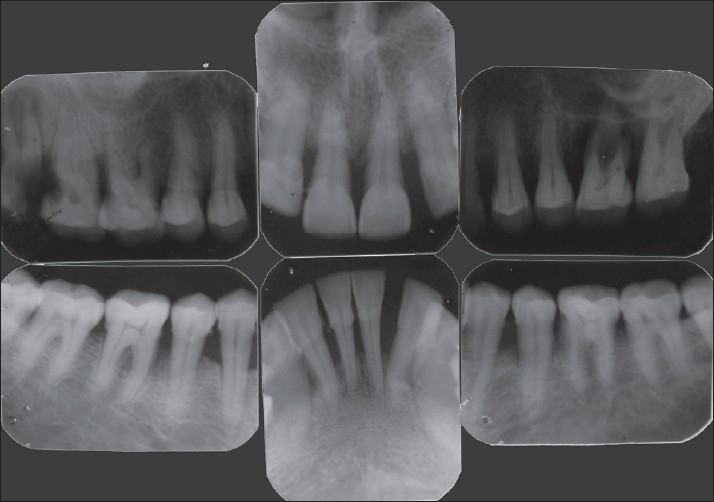
Fullmouth intraoral periapical radiographs

Intra-oral periapical radiograph showed generalized bone loss, periapical radiolucency with 26 and proximal caries with 17 and 18 [Figure 2].

### Provisional diagnosis

Based on clinical presentation and past history- Amlodipine Influenced Gingival Enlargement.

### Management

Referral to the physician: Amlodipine 2.5 mg/day replaced with Atenolol 50 mg/day.

**Figure 3:**
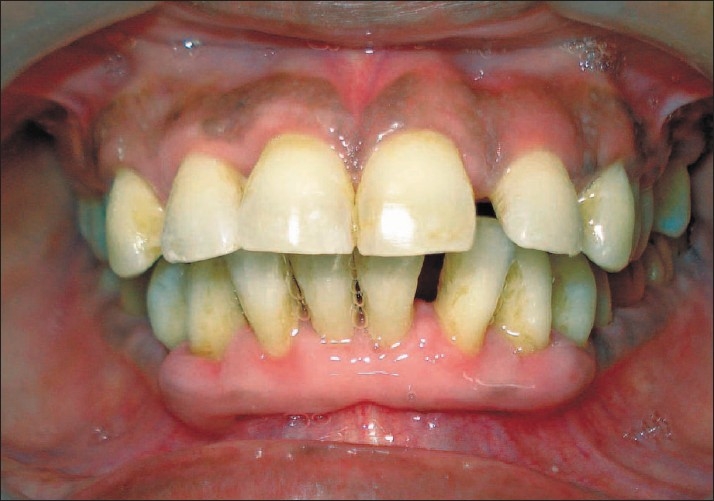
Front view 6 months after phase I therapy
- The remaining localized enlargement was removed by gingivectomy and the gingival contour was restored to healthy and maintainable state [Figure 4].

- Preliminary phase: Extraction of 18 and 26.
- Phase I therapy: Scaling and root planning, along with meticulous oral hygiene maintenance (including 0.2% chlorhexidine use) by the patient. Amalgam restoration with 17.
- Maintenance phase: For six months.
- After six months there was a marked reduction in the enlargement, except in the lower anterior region. At this time, the mean probing depth was 2.4 mm, mean plaque index was 0.6 and mean gingival index was 0.7 [Figure 3].

**Figure 4:**
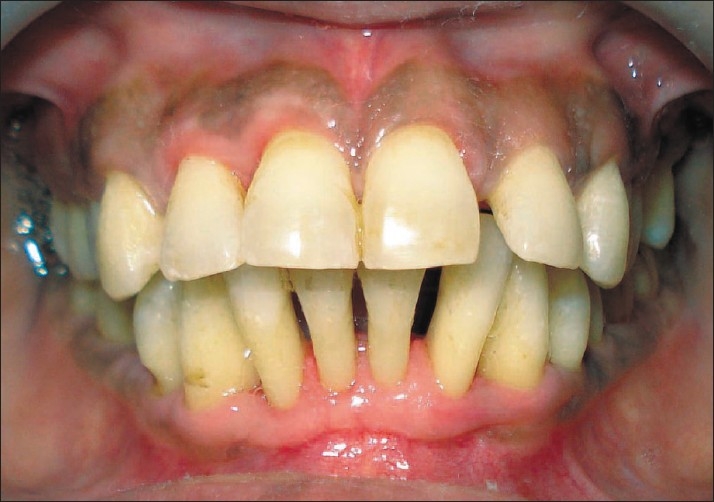
Front view 1 month after surgical therapy
- The patient was advised to maintain meticulous oral hygiene and follow recall visits.

### Histopathology

**Figure 5:**
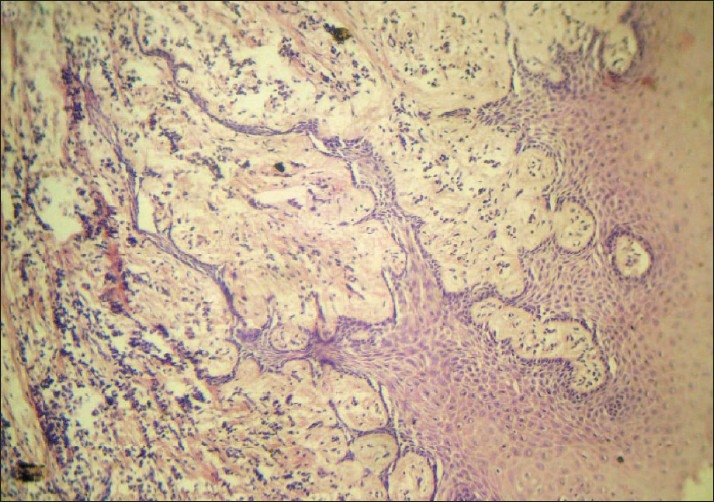
Histopathological examination

Microscopic inspection of the gingival biopsy specimens demonstrated a connective tissue hyperplasia, proliferation of overlying epithelium, and elongated reteridges penetrating the connective tissue together with few inflammatory cells [Figure 5].

### Final diagnosis

The lesion was diagnosed as Amlodipine Influenced Gingival Enlargement based on clinical and histopathological evidences.

## DISCUSSION

Gingival enlargement, with its potential cosmetic implications and also providing new niches for the growth of microorga-nisms is a serious concern for both the patients and clinician.

Amongst several pathogenesis of drug influenced gingival enlargement the plaque induced inflammatory changes is pivotal. The nature of the relationship between plaque and the expression of gingival enlargement is unclear.[6–9] In the present report, a marked reduction of the enlargement occurred following scaling and root planning. This goes in accordance with the findings of Hancock R.[10]

The difference in the occurrence of enlargement between nifedipine and amlodipine is of interest, since both drugs are dihydropyridines and hence structurally similar. However, two drugs differ in a way that, amlodipine is more polar than other dihydropyridines, with pKa value 8.7. Thus the drug may not pass through cell membrane without an active transport mechanism. By contrast, nifedipine is intensely lipophillic and will dissolve readily within the cell membrane and pass into the cytoplasm. The majority of amlodipine will be tissue bound (and hence “inactive”) rather than circulating freely in the blood.[2]

In the present case, the firm growth remained in the lower anterior region, because of: i) inadequate plaque control in this region due to protruding gingival morphology and teeth malpositioning ii) the flat surface of lower anterior teeth led to improper food shedding iii) the enlargement was more in the lower anterior region at baseline.

A significant reduction occurred after conservative treatment hence, the need of surgery was limited.

## CONCLUSION

The use of medications with the potential to contribute to the development of gingival enlargement is likely to increase in the years to come. Whenever possible, treatment should generally target on drug substitution and effective control of local inflammatory factors such as plaque and calculus. This is the most practical and effective way to control the recurrence of enlargement. When these measures fail to cause resolution of the enlargement, surgical intervention is recommended.
